# Automatic diagnosis of retinoblastoma and quantitative analysis of retinal vascular morphology using deep learning

**DOI:** 10.3389/fcell.2026.1962785

**Published:** 2026-09-22

**Authors:** Xinyi Deng, Hui Liu, Yijing Chen, Lijun Shen, Lingzhi Cai

**Affiliations:** 1 Center for Rehabilitation Medicine, Department of Ophthalmology, Zhejiang Provincial People’s Hospital (Affiliated People’s Hospital, Hangzhou Medical College), Hangzhou, Zhejiang, China; 2 Department of Precision Machinery and Instrumentation, University of Science and Technology of China, Hefei, China; 3 Department of Breast Surgery, Hangzhou First People’s Hospital, Hangzhou, Zhejiang, China

**Keywords:** artificial intelligence, deep learning, eye tumor, retinal vascular morphology, retinoblastoma

## Abstract

**Purpose:**

To develop a convolutional neural network-based artificial intelligence identification diagnostic system for ultra-wide field fundus images of retinoblastoma (RB) and quantify its vascular morphology features.

**Methods:**

A retrospective study. 183 ultra-wide field fundus images from 54 patients diagnosed with RB were included, along with 250 images of healthy control. Of these, 277 images were used as the training set, 100 images were used as the validation set, and 56 images were used for the performance test. A deep learning based on DenseNet was used to train the AI auto-diagnostic network for RB. After manual labelling to remove the lesion area, the binary skeleton vessels of the retina were automatically extracted and segmented. Vascular morphological characteristics were subsequently assessed quantitatively using the corresponding mathematical model.

**Results:**

The diagnostic performance of the network was evaluated using sensitivity, specificity and F1 score, respectively. In the validation set, the automated diagnostic system achieved 0.98 sensitivity, 0.94 specificity, and 0.96 F1 score. In the test set, the system achieved 0.93 sensitivity, 0.79 specificity, and 0.87 F1 score. In quantitative vascular morphology analysis, compared with the healthy control group, the vascular angle of RB patients was increased, while the vascular density, fractal dimension, and number of vascular branches were decreased (all P < 0.001).

**Conclusion:**

In this study, we developed an artificial intelligence system for automatic identification and diagnosis of RB in ultra-wide field fundus imaging, and were able to automatically and quantitatively analyze the retinal vascular morphological features of patients with RB.

## Introduction

Retinoblastoma is one of the most common primary intraocular tumors in children, with a global incidence of 1/15,000-20,000 (Kivela, 2009). Retinoblastoma develops as the result of mutations in the RB gene (Helen et al., 2008), which may lead to severe visual impairment, high rates of blindness, and even life-threatening conditions if tumor metastasis or spread occurs in advanced stages. The detection and early treatment are the key to improving survival and saving eyes and vision (Dimaras et al., 2015).

Approximately 11% of all RB children live in high-income countries, approximately 69% in middle-income countries, and approximately 20% in low-income countries (Dimaras et al., 2015). Despite the higher prevalence in low- and middle-income countries, most RB treatment centers are concentrated in developed countries, with estimated mortality rates of 60%, 33% and 21% in low-, lower-middle- and upper-middle-income countries, respectively (Carlos et al., 2002; Chawla et al., 2015; Masood, 2012). This is in sharp contrast to the 3–5% mortality rate in Europe, Canada and the United States (Kivela, 2009; Dimaras et al., 2012; MacCarthy et al., 2006; Nyamori et al., 2012; Leal-Leal et al., 2004). A global cross-sectional study in 2017 has reported that more than half of the global RB patients are from Asia (Ido Didi et al., 2020). The estimated number in China was 1,103 and 911 in 2013 and 2023, respectively (Rustam and Tero, 2015).

The neonatal fundus imaging system based on wide-field digital retinal imaging provides prompt detection of neonatal-associated fundus disorders such as RB. However, the inadequacy of specialists in pediatric ophthalmology is a major concern in developing countries, where the screening of eye diseases requires enormous effort, both human and financial, from the healthcare system. With the introduction of the ultra-wide field Scanning Laser Ophthalmoscope (SLO), which can capture up to 200° of the retina (covering most of the fundus) in a single, non-invasive shot. In this case, the peripheral retina can be screened without the inconvenience of dilating the pupils (Witmer et al., 2013; Nagiel et al., 2016). This screening equipment has been widely used for the diagnosis as well as the follow-up of various fundus diseases (Nagiel et al., 2016; Hideharu et al., 2017), including screening for RB. However, there are still a relatively large number of physicians who may have a lack of knowledge regarding fundus imaging of RB, failing to diagnose it in a timely and accurate manner. Hence, automatic identification and diagnosis of RB in ultra-wide field images by artificial intelligence has become a clinical necessity.

Deep learning algorithms have recently achieved remarkable performance in the application of complicated medical image analysis, with much research reported on automated diagnosis of fundus diseases such as retinopathy of prematurity, diabetic retinopathy, age-related macular degeneration, etc. (Mao et al., 2020a; Brown et al., 2018; Ting et al., 2018). Several studies have also explored AI-based automated diagnosis of RB. Vempuluru et al. developed an AI model for RB detection and International Classification of Retinoblastoma (ICRB) grouping in a multiracial cohort of 2,473 images, achieving a sensitivity of 93% and specificity of 96% for RB detection (Vempuluru et al., 2024). Cruz-Abrams et al. distinguished RB from pseudo-retinoblastoma using machine learning with a sensitivity of 98.6% and specificity of 97.0% (Cruz-Abrams et al., 2025). A recent systematic review and meta-analysis by Lima et al. reported a pooled sensitivity of 98.2% and specificity of 98.5% for AI-based RB detection across 9,902 fundus images (Lima et al., 2025). However, there have few reports of RB automatic diagnosis using ultra-wide field imaging. In this study, we implemented an AI-automated RB diagnosis based on the DenseNet convolutional neural network (CNN) algorithm using a relatively small number of image samples and assessed its performance. In addition, we further employed the network to perform automated segmentation of retinal vasculature in RB and analyzed the morphological characteristics of retinal vasculature in RB.

## Materials and methods

### Participants

The patients included in this study were from Zhejiang Provincial People’s Hospital and Eye Hospital of Wenzhou Medical University. The study adhered to the Declaration of Helsinki, and written informed consent was obtained from the guardians. The diagnosis of RB was confirmed by the ophthalmologist through ultrasound of the eye, fundus photography or CT. A total of 183 retinal images from 54 RB patients and 250 images from healthy participants were collected between November 2017 and October 2024 using the ultra-wide field imaging system (Optos, United Kingdom), under the standard manufacturer-recommended protocol (capturing up to 200° of the retina without pupil dilation). Images with unsatisfactory quality due to significant refractive media opacity and diffuse lesions were excluded. Each image in the dataset was classified as present or absent of RB based on the clinical diagnosis. In addition, to ensure the accuracy of the system’s diagnosis, all anonymized images were independently reviewed by 2 retinal specialists, and a final diagnosis of the image was given only when the diagnoses of the 2 retinal specialists were in agreement. To train the RB segmentation network, the RB images were manually labeled. The dataset was divided at the patient level to prevent data leakage: the 54 RB patients were randomly partitioned into three independent groups, and all images from each patient were assigned exclusively to either the training, validation, or test set. The final dataset comprised 277 images for the training set, 100 images for the validation set, and 56 images for the test set.

### Network architecture and training

This study employed deep learning methods based on DenseNet (Figure 1), which was constructed by concatenating multiple dense blocks. Within each dense block, there were several convolutional combinations, and the input of each convolutional combination was the concatenation of all the feature maps from the previous outputs. Each convolutional combination consisted of a 1 × 1 convolutional layer stacked with a 3 × 3 convolutional layer, forming a bottleneck structure. Adjacent dense blocks were connected by transition layers, which were composed of a 1 × 1 convolution and a 2 × 2 average pooling. The former reduced the number of channels of the input feature map by half, while the latter halved the height and width dimensions of the feature map. DenseNet adopted a dense connectivity mechanism, where all layers are connected to each other. Each layer was concatenated with the previous layers along the channel dimension, enabling feature reuse as the input for the next layer. This not only alleviated the problem of vanishing gradients but also allowed DenseNet to achieve better performance than ResNet with fewer parameters and computations. The bottleneck and transition layer improved computational efficiency and compressed the parameter volume.

**FIGURE 1:**
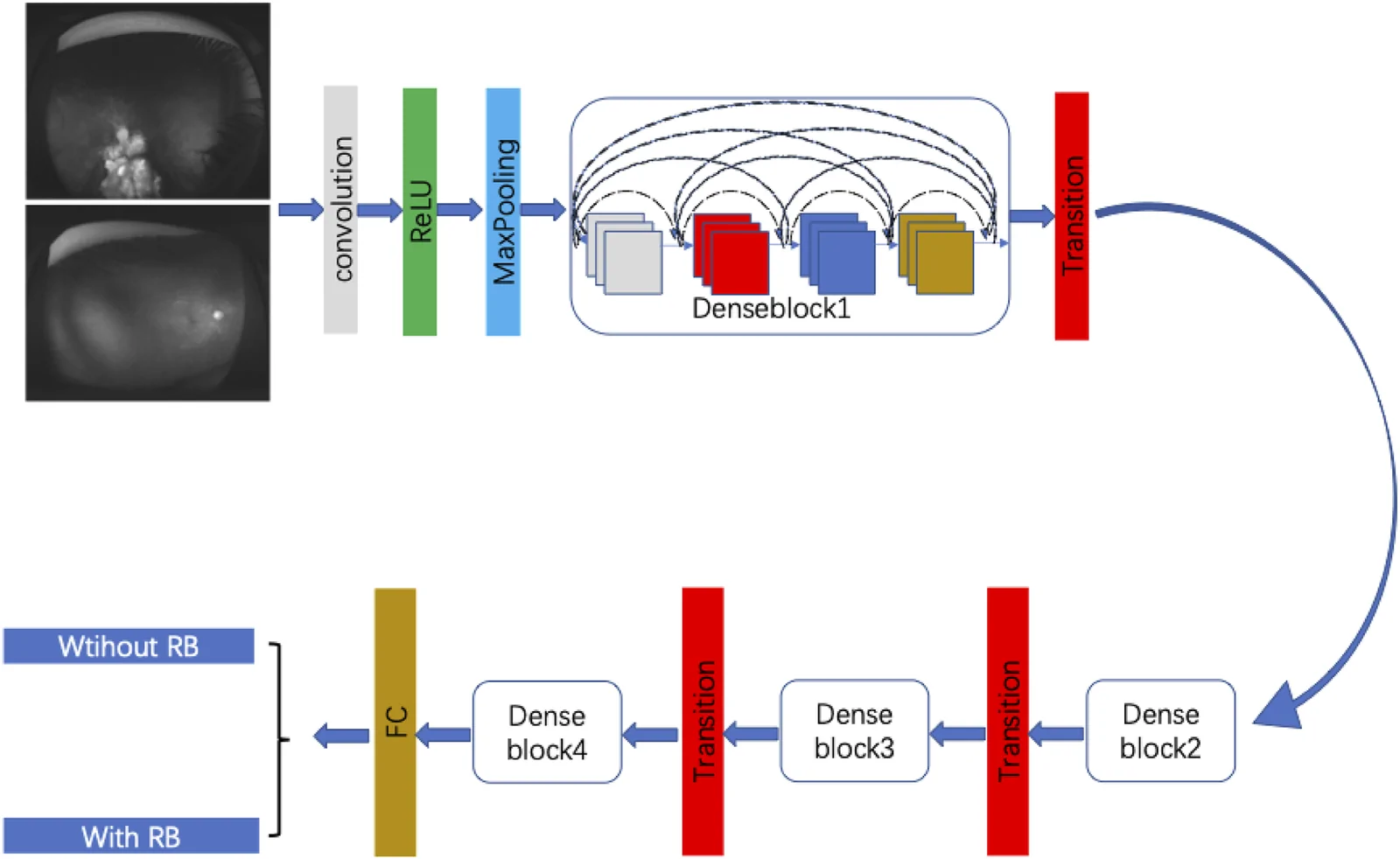
Schematic diagram of the DenseNet network.

### Quantitative vascular analysis

The binarized skeleton retinal vessels were extracted after the lesion area was manually labeled and covered. Morphological characteristic parameters of vessels, including vessel angle, fractal dimension, vessel density, and number of vessel bifurcation points were assessed. The vessel angle was measured by connecting the macular center and the optic disc as the reference axis and calculating the angle of each vessel to it. Fractal dimension was calculated on the basis of binarized vessels using the standard box counting method proposed by Stosic and Mainster (Stosic and Stosic, 2006; Mainster, 1990), which provided an overview of the branching pattern of the retinal vascular tree, with larger values indicating a more complex vascular pattern (Liew et al., 2008). Vessel density was defined as the ratio of vascular area to the entire image area after removing the optic disc and lesion area. The number of vascular branches was calculated by counting the number of pixels in the connected domain, reflecting the geometric complexity of the vessels. Figure 2 presents typical retinal and its vessel segmentation images of healthy controls and RB patients (the blood vessels in the tumor region were manually removed in the subsequent vascular quantification analysis).

**FIGURE 2:**
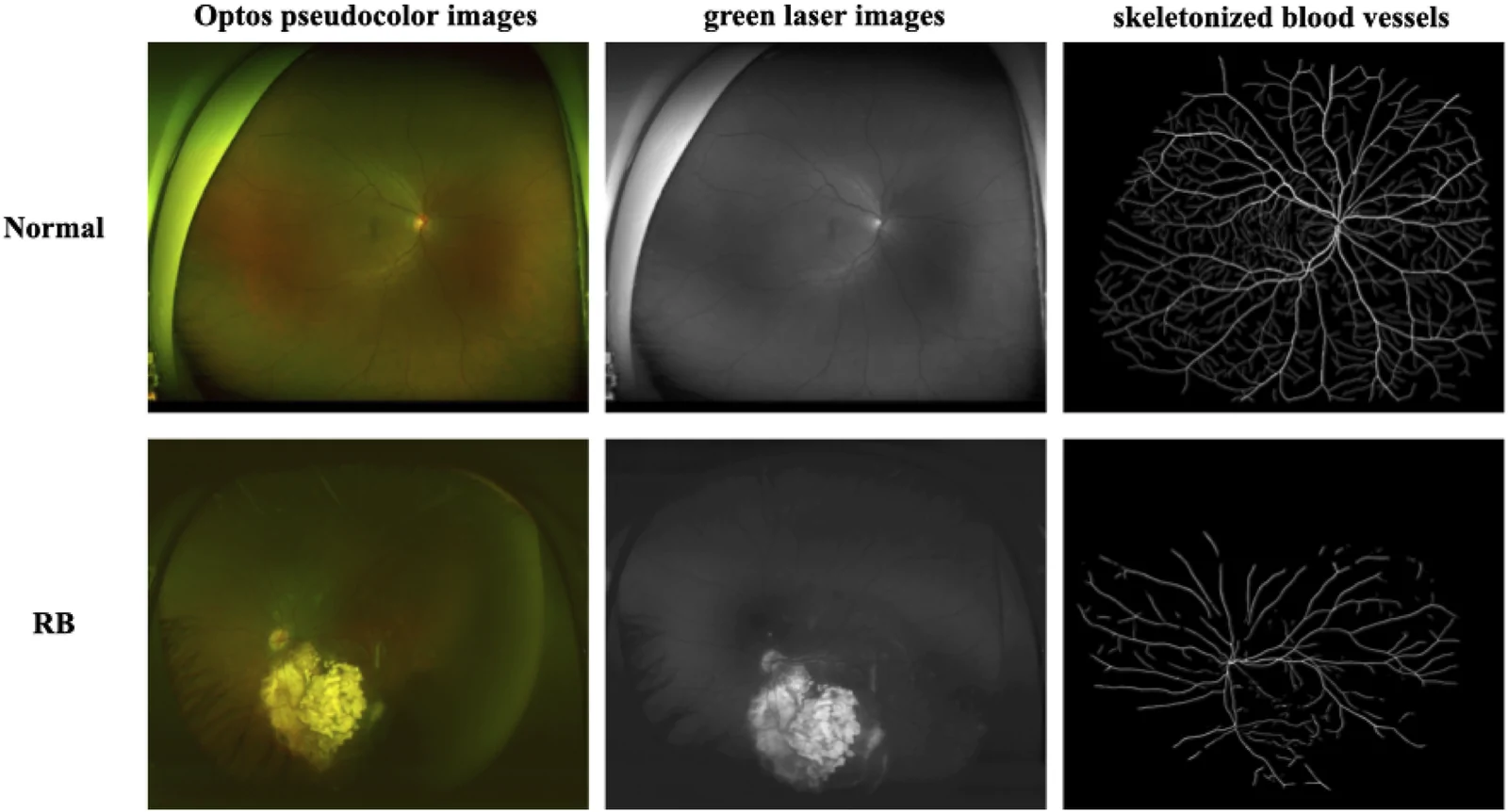
Schematic diagram of healthy control and RB eyes and corresponding skeletonized retinal vessels.

### Statistical analysis

Statistical analyses were performed using SPSS software (version 26.0). Quantitative data were expressed as mean ± standard deviation, and one-way ANOVA was used for comparison between groups; the chi-square test was used for comparison of count data. P values < 0.05 were considered to be statistically significant.

## Results

### Performance of segmentation system

The segmentation network was trained to perform two tasks: retinal vessel segmentation and optic disc segmentation. For optic disc segmentation, the network achieved 0.94 sensitivity, 0.99 specificity, and 0.94 F1 score, respectively. For retinal vessel segmentation, the performance of the network for segmentation in the validation set achieved 0.72 sensitivity, 0.99 specificity, and 0.73 F1 score, respectively.

### Performance of the prediction system

The diagnostic performance of the automated RB identification system was evaluated on both the validation set and the test set. In the validation set, the system achieved 0.98 sensitivity, 0.94 specificity, and 0.96 F1 score, with a false negative rate of 2% and a false positive rate of 6% (shown in Table 1).

**TABLE 1:** Predictive performance in the validation set.

​	Predicted value		​
True value	Negative	Positive	Total
Negative	47	3	50
Positive	1	49	50
Total	48	52	​

Positive: retinoblastoma; negative: healthy control. Sensitivity = 0.98, specificity = 0.94, F1 = 0.96.

In the test set, the automated diagnostic system achieved 0.79 specificity, 0.93 sensitivity, and 0.87 F1 score, respectively, with the false negative rate of 7.14% and the false positive rate of 21.43% (shown in Table 2).

**TABLE 2:** Predictive performance in the test set.

​	Predicted value		​
True value	Negative	Positive	Total
Negative	22	6	28
Positive	2	26	28
Total	24	32	​

Positive: retinoblastoma; negative: healthy control. Sensitivity = 0.93, specificity = 0.79, F1 = 0.87.

### Quantitative vascular analysis

Compared with healthy controls, children with RB had increased vessel angles and decreased vessel density, fractal dimension, and number of vascular branches (all P < 0.001, shown in Table 3).

**TABLE 3:** Vascular characteristics between children with RB and healthy control.

Characteristics	RB	Healthy control	P-value
Vessel angle (°)	37.79 ± 5.04	31.57 ± 2.68	<0.001
Df	1.28 ± 0.10	1.42 ± 0.43	<0.001
Vessel density (%)	0.78 ± 0.45	3.61 ± 1.12	<0.001
Number of vascular branches	820.2 ± 803.2	7784.5 ± 3098.0	<0.001

Df, fractal dimension.

To address the potential effect of tumor exclusion on the number of vascular branches, a supplementary analysis was performed. For each RB eye, an equidistant ROI was delineated at the mirror-image symmetrical position in the quadrant containing the tumor, with the optic disc as the center. The number of vascular branches within this region was calculated and used as a correction value for the lesion area. The corrected number of vascular branches remained significantly lower in RB eyes than in the controls (P < 0.001; Supplementary Table S1).

## Discussion

This study was based on the ultra-wide field fundus imaging system, combined with a deep learning model, to construct a feature segmentation and identification system for RB imaging and to quantitatively analyze the retinal vascular morphological features of children with RB, achieving automatic diagnosis and screening for RB.

In recent years, artificial intelligence and deep learning techniques have been extensively employed in the characterization and screening for a variety of pediatric vision-impairing eye diseases, including retinopathy of prematurity (Mao et al., 2020a) and familial exudative retinopathy (Ye et al., 2020). And as the most malignant intraocular tumor in infancy and childhood, RB, if not diagnosed in time and treated appropriately, often leads to serious consequences, ranging from removal of the eyeball to extraocular dissemination and even life-threatening outcomes (Rusakevich and Schefler, 2022). The survival rate of children with RB is significantly lower in less developed countries than in developed countries due to lack of timely diagnosis (AlAli et al., 2017).

The binocular indirect ophthalmoscope is the golden standard for the diagnosis of RB, which is usually performed under anesthesia in children by examining the entire retina through a scleral depressor. Combined with fundus photography, ultrasound, and orbital MRI, RB was diagnosed and then staged. However, only a few pediatric ophthalmologists are currently skilled in binocular indirect ophthalmoscopy, which is one of the main reasons for the lack of early RB diagnosis in China. Therefore, it is necessary to build a simple, effective, and reliable artificial intelligence diagnosis system for RB.

At present, AI-automated diagnostic systems for RB were rarely reported. Two smartphone apps, MDEyeCare and CRADLE, based on the detection of leukocoria by ocular red-light reflex in digital photography, were effective in improving the detection rate of RB (Khedekar et al., 2019). Bernard et al. developed the EyeScreen system based on the aforementioned system, combined with the machine learning model for automatic identification of leukocoria, achieving a sensitivity of 87% and a specificity of 73% (Bernard et al., 2022). However, although some studies have shown that leukocoria could also present in the early stages of RB, its prevalence was low (Abdolvahabi et al., 2013). Therefore, the automatic diagnostic system for RB, by detecting leukocoria by digital photographic techniques, had a low detection rate, which was often at an advanced stage by the time it was detected, resulting in failure of the optimal treatment. In addition, detection approaches through red-light reflection often require infants and children to stare at specific directions to be photographed, which is one of the limitations in practical application.

Ultra-wide field fundus photography has a wide imaging range and would cover 80% of the retinal area without pupil dilation and eye rotation, which greatly improves the detection rate of retinal disorders and reduces the risk of missed diagnosis. Artificial intelligence automated diagnostic systems using ultra-wide field fundus imaging have been used in a variety of eye diseases (Cao et al., 2022). In addition, ultra-wide field fundus imaging with ROP has been shown to be safe and effective in infants and children (Mao et al., 2020b; Patel et al., 2013). In this study, we achieved automatic identification and diagnosis for RB in ultra-wide field fundus imaging. In the validation set, the system achieved a sensitivity of 98%, a specificity of 94%, and an F1 score of 96%. In the test set, the system achieved 86% accuracy, 93% sensitivity, 79% specificity, 7.14% false-negative rate, and 21.43% false-positive rate. Due to the rarity and severity of RB, prioritizing sensitivity is clinically preferable to minimize missed diagnoses. Further, a sensitivity-prioritized threshold could be achieved by lowering the decision threshold, which may increase false positives but is clinically acceptable in screening contexts.

In this study, quantitative vascular analysis excluded the tumor region. It should be noted that retinoblastoma is a solid, vascularized tumor with spatially heterogeneous vessel distribution, the center of which contains necrotic regions with sparse vessels. Therefore, to ensure the analysis focused on comparable retinal areas across patients, all vascular measurements were performed on the preserved retinal vasculature in regions unaffected by the tumor (Folberg et al., 2000; Piña et al., 2009). Studies in children with RB using Optical Coherence Tomography Angiography (OCTA) have shown unique vascular morphological characteristics, such as disorganized and complex vascular branching patterns, irregular vascular caliber, and vascular dilatation and tortuosity (Fernandez et al., 2021). In addition, the reduction in the deep retinal vascular density has been reported in the macular region (Chen et al., 2023; Sioufi et al., 2019). Although the ROI and measurement methods differ, the results of the present study were consistent with those findings. One possible explanation was the minimal toxicity associated with intravenous chemotherapy in RB, ischemic changes induced by chemotherapy may lead to pathological changes in the vasculature, which may further affect vascular morphological changes.

One of the limitations of this study was the small number of RB patients from a single center. Due to concerns regarding image quality and segmentation performance, patients with severe RB were excluded because the vascular imaging in their retinal images was unclear. In addition, quantitative analysis of retinal vessels excluding the tumor area may introduce bias. Future studies with larger sample sizes and standardized fixed ROI would help in improving the RB automated diagnostic performance and vascular analysis. With the emergence of portable wide-field imaging devices and the adaptation of algorithms through transfer learning, our method would become easier to apply in resource-constrained environments in the future.

In conclusion, based on ultra-wide field fundus imaging, combined with deep learning technology, it is expected to construct an automatic diagnosis system of fundus diseases in infants and children with multiple diseases in the future, which would greatly reduce the burden of diagnosis and treatment of eye diseases in infants and children.

## Data Availability

The original contributions presented in the study are included in the article/Supplementary Material, further inquiries can be directed to the corresponding author.
