# Supplementary material for: Automatic diagnosis of retinoblastoma and quantitative analysis of retinal vascular morphology using deep learning
Source: Front Cell Dev Biol. 2026 Sep 22;14:1962785. doi: 10.3389/fcell.2026.1962785 (PMC13638580; doi:10.3389/fcell.2026.1962785)
Supplement: Supplementary file 1 [file Table1.docx]

**Supplementary Table 1**

Comparison of the number of vascular branches before and after correction with healthy control

| Number of vascular branches | RB | healthy control | P-value |
| --- | --- | --- | --- |
| Original reported value | 820.2±803.2 | 7784.5±3098.0 | <0.001 |
| Corrected value | 974.9±914.3 | 7784.5±3098.0 | <0.001 |
